# Human-Mediated Land Use Change Drives Intraspecific Plant Trait Variation

**DOI:** 10.3389/fpls.2020.592881

**Published:** 2021-01-14

**Authors:** Hayley Schroeder, Heather Grab, André Kessler, Katja Poveda

**Affiliations:** ^1^Department of Entomology, Cornell University, Ithaca, NY, United States; ^2^School of Integrative Plant Sciences, Cornell University, Ithaca, NY, United States; ^3^Department of Ecology and Evolutionary Biology, Cornell University, Ithaca, NY, United States

**Keywords:** microevolution, natural selection, plant secondary metabolites, floral traits, plant fitness, plant defense, landscape simplification, plant–insect interactions

## Abstract

In the Anthropocene, more than three quarters of ice-free land has experienced some form of human-driven habitat modification, with agriculture dominating 40% of the Earth’s surface. This land use change alters the quality, availability, and configuration of habitat resources, affecting the community composition of plants and insects, as well as their interactions with each other. Landscapes dominated by agriculture are known to support a lower abundance and diversity of pollinators and frequently larger populations of key herbivore pests. In turn, insect communities subsidized by agriculture may spill into remaining natural habitats with consequences for wild plants persisting in (semi) natural habitats. Adaptive responses by wild plants may allow them to persist in highly modified landscapes; yet how landscape-mediated variation in insect communities affects wild plant traits related to reproduction and defense remains largely unknown. We synthesize the evidence for plant trait changes across land use gradients and propose potential mechanisms by which landscape-mediated changes in insect communities may be driving these trait changes. Further, we present results from a common garden experiment on three wild *Brassica* species demonstrating variation in both defensive and reproductive traits along an agricultural land use gradient. Our framework illustrates the potential for plant adaptation under land use change and predicts how defense and reproduction trait expression may shift in low diversity landscapes. We highlight areas of future research into plant population and community effects of land use change.

## Introduction

Agriculture now represents the largest anthropogenic biome on the planet, occupying over a third of the earth’s ice-free land (Foley et al., 2005; Ellis and Ramankutty, 2008; Holzschuh et al., 2011; Seto et al., 2011). Loss of natural habitats is linked to rapid declines in biodiversity worldwide, driving many species locally extinct and homogenizing species composition (Brooks et al., 2002; Keil et al., 2015). Precipitous declines in insect abundance and diversity documented in the last century are largely attributed to habitat loss, fragmentation, and degradation associated with land use change (Hallmann et al., 2017; Jauker et al., 2019; Seibold et al., 2019). Though insect abundance and diversity is generally declining, insects that can utilize the modified habitat (e.g., crop pests) often undergo population surges (Chaplin-Kramer et al., 2011; Veres et al., 2013). The consequences of insect population shifts are not limited to modified landscapes. Because of their frequently high mobility, changes in insect abundance and composition within modified habitat matrices also affect species interactions in surrounding habitats as insects track resources across the landscape. Due to their central role in ecosystem functioning and species interactions, changes in insect communities therefore can have cascading effects on ecological processes within and around modified landscapes. However, the mechanisms driving the directionality and magnitude of the effects of landscape-mediated changes in insect communities on wild plants remain unclear (Irwin et al., 2018). Whether wild non-crop plants in landscapes dominated by agriculture are adapting to compensate for declines in native pollinators and natural enemies and outbreaks of insect herbivores could have important implications for wild plant population persistence (Thomann et al., 2013).

The coevolution of plants and insects has resulted in both an evolutionary arms race of defense and counter defense as well as a sweeping reliance on insects for reproduction. Given that both antagonistic and beneficial insects are major agents of selection, frequently on similar plant traits, it is likely that changes in the insect community driven by land use change alter plant trait evolution (Schiestl and Johnson, 2013). Additionally, antagonistic and beneficial insect populations may impose conflicting selection on the limited resources plants can allocate to defense and reproduction (Knauer and Schiestl, 2017). Because the combined effects of herbivores and pollinators can be reinforcing or conflicting, changes in either community could alter the outcomes of selection on morphological and phenological traits (Gómez, 2003; Kessler and Halitschke, 2009; Sletvold et al., 2015).

**FIGURE 1:**
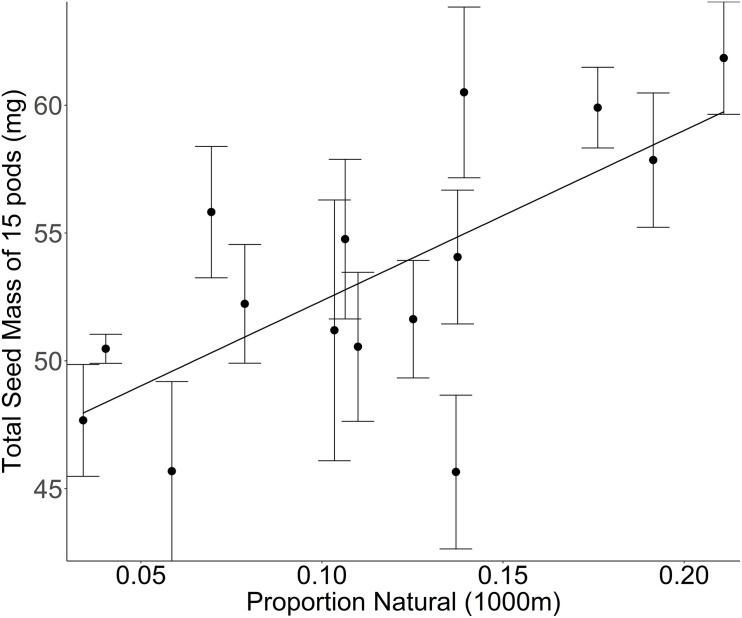
Mass of seeds produced by the first 15 self-fertilized pods on the central inflorescence of *Capsella bursa-pastoris* plants sourced from 15 sites along a gradient of increasing proportion of agricultural land cover and grown together in a greenhouse common garden [*F*_(__1_,_13__)_ = 9.4862, *p* = 0.0088]. The proportion of natural cover at a 1000 m radius around the collection site is a measure of land use composition but does not provide any information regarding the intensity of the management practices within any given land use. Mean ± 1 SE values per site are calculated based on the measurement of 201 different *C. bursa-pastoris* plants (2–25 plants per site).

Ultimately, there is evidence that landscape-mediated changes in insect communities affect fitness in wild plants (Holzschuh et al., 2011). For example, wild plants in natural habitats surrounded by landscapes dominated by natural land cover produce more and heavier seeds than those surrounded by landscapes dominated by agriculture, matching with metrics of pollinator availability within each landscape (Albrecht et al., 2007; Diekötter et al., 2010; Jakobsson and Ågren, 2014). Similarly, our data from a greenhouse common garden experiment in which shepherd’s purse plants (*Capsella bursa-pastoris*) were allowed to self-pollinate, we found that plants sourced from populations surrounded by natural land cover, produce more and/or heavier seeds than populations surrounded by more agricultural land (see Supplementary Methods). These results suggest that the self-pollination success is reduced in landscapes with increased agricultural cover, indicating either (1) a reduced ability to autonomously self-pollinate, or (2) a higher level of self-incompatibility (Figure 1). The evidence that seed number and size varies with landscape context motivates this review to examine the potential for landscape mediated adaptation in wild plants.

**FIGURE 2:**
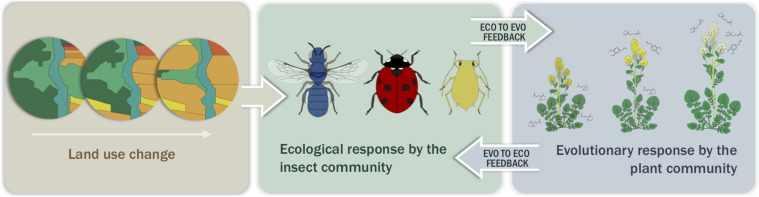
Simple conceptual model with one potential output demonstrating how land use change, through habitat loss, species introduction and/or management practices, affects the insect community, which in turn selects for certain plant traits (depicted here are plant size, flower color, and linalool production) at the population level. These changes in traits lead to an eco-evolutionary feedback loop between the insect community and the plant population.

Here we hypothesize that landscape-mediated changes in insect community composition result in predictable variation in plant traits (Figure 2). While land use change can take many forms, we focus largely on agriculture in our predictive framework because it represents the largest land area of human-mediated land use change (Ellis and Ramankutty, 2008). However, when available, we also include examples of the effects of urbanization. Further, although agriculture and urbanization can encompass many levels of intensification and a large variety of management practices, we center our work on the replacement of natural areas by agricultural or urban areas *per se.* To define underlying assumptions, we first provide evidence that herbivores, natural enemies, and pollinators are agents of selection on plant traits. Next, we outline how land use change affects each insect functional group, highlighting the potential mechanisms by which landscapes could mediate these changes and developing predictions for the outcomes of insect utilization of the habitat. Finally, we synthesize the current evidence that landscape-mediated effects on insect communities result in evolutionary changes in plant populations and supplement these data with a case study in three wild Brassicaceae species. Through this review we provide a framework to inform predictions for plant trait evolution under land use change scenarios, focusing on agriculturally driven habitat conversion. We propose that land use change gradients can serve as a model for studying microevolutionary dynamics and the evolution of species interactions by simulating the timeline of land use change experienced by plants and insects in recent history. The functional traits and natural history of the interactors will dictate the scale at which the landscape is experienced while the rate of adaptation will be dependent on the strength of selection and genetic constraints of the plant.

## Land Use Change Affects Plant Direct Defenses, Mediated by Herbivore Communities’ Responses to Land Use Change

### Herbivores Affect Plant Defenses

Plants and herbivores are thought to be locked in a co-evolutionary arms race where plant defenses counter herbivore resistance to plant defenses (herbivore counter defenses) and *vice versa* (Ehrlich and Raven, 1964). Although the major prediction of this hypothesis, the reciprocal natural selection, is rarely tested, it is increasingly clear that herbivores frequently function as major agents of selection on plant chemistry and defenses (Agrawal et al., 2012; Uesugi and Kessler, 2013; Gervasi and Schiestl, 2017; Kalske et al., 2019). Increased selection by herbivores usually results in higher mean values for constitutive plant resistance in a population (Agrawal et al., 2012; Bode and Kessler, 2012). Relief from herbivore pressure usually selects for decreased herbivore resistance and higher competitive ability (Uesugi and Kessler, 2013; Uesugi et al., 2017).

The evolution of novel defense traits is predicted to result in the plants’ escape from a significant proportion of potential herbivores available in an environment (Kessler, 2015). Similarly, the occurrence of a novel herbivore species (or genotypes) can have strong negative effects on plant communities and populations (Moxley et al., 2017). Plant traits that mediate resistance against herbivores can function directly on their attacker or indirectly mediate interactions with third party organisms [e.g., natural enemies of herbivores (Dicke and Baldwin, 2010; Kessler and Heil, 2011), associational resistance (Barbosa et al., 2009), keystone herbivores (Poelman and Kessler, 2016)] to the plants’ advantage. Direct defenses can be physical structures (e.g., thorns, spines, prickles, trichomes, silica, and thickened cell walls), chemical or combinations of the two (e.g., exudating trichomes, latex, adhesives). In theory, these traits can occur simultaneously in a plant but may differentially affect different attackers. Consequently, the way in which plant defenses are altered in response to natural selection strongly depends on the major agent of selection as well as the ecological context in which the interactions are played out. For example, general relief from herbivory in tall goldenrod, *Solidago altissima*, populations resulted in lower mean resistance to a major specialist herbivore, the goldenrod leaf beetle, *Trirhabda virgata*, but had no effect on the resistance to an herbivore uncommon in this system, the fall armyworm (*Spodoptera frugiperda)* (Bode and Kessler, 2012). Nevertheless, if land use changes are associated with significant shifts in the herbivore community (diversity, abundance, and composition), plant population genetic changes and shifts in the mean defense phenotypes can be predicted.

Plants respond to environmental stressors, such as herbivory, with an alteration of their metabolism (Karban, 2011). Such plant induced responses to herbivory can function in multiple different ways: (I) Induced changes to primary and secondary metabolism allow the plant to adjust to exposure to stress and thus maintain structural integrity and reproduction (tolerance). (II) Some metabolic responses to herbivory include the increased production of toxic, antidigestive or antinutritive compounds and so confer increased resistance when needed, in case of an actual attack (induced defenses) (Kessler and Baldwin, 2002). (III) The simple change in metabolism *per se*, rather than the increased production of defensive compounds can provide a moving target for foraging herbivores (moving target hypothesis) (Adler and Karban, 1994). (IV) Herbivore-induced changes in secondary metabolism can provide information about the physiological, attack and defense status of the plant and so mediate interactions with natural enemies of their herbivores (indirect defenses) (Dicke and Baldwin, 2010) or neighboring plants (associational resistance) (Heil and Karban, 2010). The plant’s ability to respond to herbivory is a heritable trait and thus expected to be subject to selection by herbivory much like constitutive defensive traits discussed above.

Overall, changes in the herbivore community (abundance, diversity, composition) can have short-term ecological effects due to the inducibility of metabolic changes in response to herbivory or they can have intermediate-term microevolutionary effects on the population genetic structure that feeds back to the ecological interactions (Figure 2). Correlative studies that target the relationship between land use change and plant–organismal interactions need to consider both potential causes when studying population-wide effects.

### Land Use Change Affects Herbivores

The response of herbivore communities and populations to land use change varies dramatically. Greater proportions of agriculture in the surrounding landscape can lead to augmentation, suppression or no change in the herbivore communities or populations (Table 1). Increasing agriculture in the surrounding landscape has been shown to decrease herbivore abundance on wild plants in a series of studies (Table 1). For example, seed predators and folivores were more common on wild sunflowers (*Helianthus annuus texanus*) that were farther away from cultivated sunflowers (*H. annuus*), than those growing next to cultivated sunflowers (Chamberlain et al., 2013). Domesticated crops are often considered less defended than their wild relatives, resulting in increased herbivore damage and herbivore preference and performance on crops in comparison to a wild conspecific (Whitehead et al., 2017). In those cases where wild plants are better defended than domesticated plants, it is likely that at the landscape scale, herbivores would prefer crop-host over non-crop-hosts, this way reducing herbivore pressure on the wild plants. Additionally, pest control methods ranging from intercropping (Tamburini et al., 2020) to the use of pesticides in cultivated crops could decrease the overall abundance of herbivores, leading to reduced herbivory in adjacent wild relatives. A long term, broad scale study in China found that the increased spatial and temporal use of Bt cotton did not just reduce the pest pressure of cotton bollworm (*Helicoverpa armigera*) on cotton, but also on other crops such as corn, peanuts and other vegetables attacked by this pest (Wu et al., 2008). This suggests that management practices aimed at decreasing pest pressure in one crop can not only affect herbivore pressure on other crops, but could also decrease pest pressure on wild plants, in those cases where herbivores are shared.

**TABLE 1:**
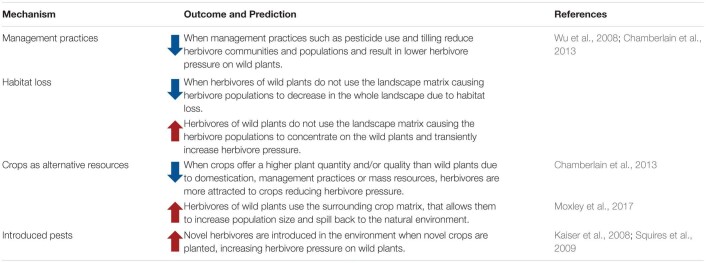
Effect of land use change on herbivore communities and populations of natural plant systems.

*Here we present the direction of the response (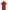 = positive effects on herbivores, 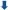 = negative effects on herbivores) and the potential mechanisms for that response. When examples from the literature are available that suggest those mechanisms, we cite them under references. Rows without references reflect areas of future work.*

Conversely, there is evidence that herbivore populations can be subsidized by agriculture and spillover to natural plant communities (Blitzer et al., 2012). The introduction of novel herbivores to a system through the use of non-native crops (Kaiser et al., 2008; Squires et al., 2009) may drive increases in herbivore pressure on wild plants. For example, the introduced coffee berry moth (*Prophantis smaragdina*), which is a known pest of coffee was found to be feeding on the wild rubiaceae, *Bertiera zaluzania*, with negative effects on the plant’s fitness (Kaiser et al., 2008). Additionally, agricultural systems provide alternative resources for existing herbivores increasing their populations (Gladbach et al., 2011). In a study done on wild sunflowers in prairie remnants, McKone et al. (2001) found that close proximity to maize fields increased the amount of corn rootworm beetles (*Diabrotica barberi* and *D. virgifera*) in wild sunflowers. The wild sunflowers had more floral damage and decreased seed set when they were close to the edges of the corn fields, due to a combination of the life history of the pest and the timing of the crop. When the adults emerge in July they feed on corn silks and immature ears, but once the crop is mature and dry they no longer prefer it and will shift to the wild sunflowers that are blooming at that time. Similarly, wild mustard (*Sinapis arvensis*) experiences higher herbivory from the pollen beetle (*Meligethes aeneus*), a pest of canola (*Brassica napus*), immediately following canola bloom in areas with higher canola cover (Gladbach et al., 2011).

Overall, land use change has a significant impact on herbivores attacking wild plants, through changes in the composition (richness, evenness, and abundance) but also very likely through changes in herbivore traits, that have not been as clearly documented (but see Gámez-Virués et al., 2015). However, the direction and severity of the impact is dependent on the management practices used in the modified habitats, the resistance traits of crops and wild plants alike and the ability of invasive herbivores that come with novel crops to attack native plants. Further, resistance in the crops and wild plants can feedback to select for resistance in the pest species. Also, indirect effects mediated by changes in the natural communities (discussed further down) could affect herbivore pressure in wild plants. Either direction, the consequences of herbivore shifts due to land use change have been shown to affect plant fitness and therefore should have consequences on plant defense traits that will ultimately allow plants to adapt to the new environment they are experiencing.

### Land Use Change Affects Wild Plant Direct Defenses

**FIGURE 3:**
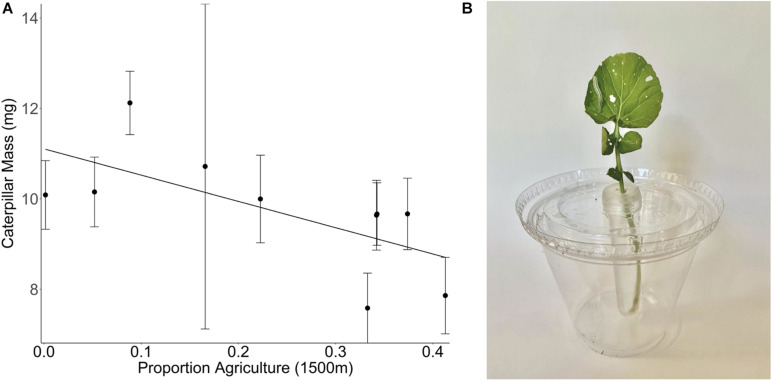
**(A)** Effect of the proportion agriculture at 1500 m on the mass of *Trichoplusia ni* caterpillars that have been feeding on *Barbarea vulgaris* plants for 3 days [*F*_(__1_,_8__)_ = 5.21, *p* = 0.0518]. *B. vulgaris* plants were collected at 10 different sites that vary in their landscape composition. The proportion of agricultural cover at a 1500 m radius around the collection site is a measure of land use composition but does not provide any information regarding the intensity of the management practices within any given land use. Mean ± 1 SE values per site are calculated based on caterpillar growth on individual severed leaves from 1 to 10 different *B. vulgaris* plants per site, with two leaf samples (and therefore two caterpillars) per plant. **(B)** Experimental set up with *T. ni* caterpillar feeding on a *B. vulgaris* leaf.

We are not aware of any studies looking at the effect of land use change on wild plant defenses. However, here we present results from our own work that has explored the importance of land use change on plant defenses. In the summer of 2017, we collected seeds of the Brassicaceae species *Barbarea vulgaris* from sites of varying landscape composition across the Finger Lakes region in New York State, United States. Plants were grown in a greenhouse common garden and bioassays were conducted with 3-day old *Trichoplusia ni* (Noctuidae) caterpillars to evaluate resistance on 3-week-old plants (see Supplementary Methods). Caterpillars gained less weight on plants sourced from sites with a higher proportion of agriculture in the surrounding landscape than those feeding on plants sourced from low agriculture sites (Figure 3). This pattern suggests that wild *B. vulgaris* plants growing in high agriculture landscapes invest relatively more in plant defenses, providing evidence for landscape mediated trait adaptation either through genetic changes in plant defense trait expression or maternal effects. More studies like this are needed to determine if this is a generalizable pattern, if wild plant adaptive responses are highly variable by species and region, and to evaluate the role of maternal effects.

## Land Use Change Affects Floral Traits, Mediated by Pollinator Communities’ Responses to Land Use Change

### Pollinators Affect Plant Traits

Flowering plants and insect pollinators share a long coevolutionary history in which plant fitness is highly dependent on the ability to attract effective pollinators. As mediators of plant reproduction, pollinators are important in shaping floral morphology, scent, and outcrossing potential. Pollinator declines can intensify selection on floral traits like flower number, size and selfing rate (Brys and Jacquemyn, 2012; Panique and Caruso, 2020). In a study that compared floral traits across time from a site with known pollinator declines, contemporary populations had larger floral displays and longer receptivity compared to their ancestor population (Thomann et al., 2015). Similarly, Castellanos et al. (2019) demonstrate that stable pollinator environments can stifle current evolutionary change while maintaining heritable variation necessary for future adaptation.

The preferences and morphology of the local pollinator communities can also drive changes in plant reproductive traits. Pollinators often prefer larger flowers (Galen, 1989; Benitez-Vieyra et al., 2006; Sandring and Ågren, 2009; Parachnowitsch and Kessler, 2010) and floral scents that are distinct and reliable within species (Parachnowitsch et al., 2012; Ramos and Schiestl, 2019). In *Erysimum mediohispanicum*, spatial variation in pollinator community assemblage resulted in divergent selection on corolla shape and tube width (Gómez et al., 2009). Nagano et al. (2014) demonstrated that floral size variation across mountain ecosystems was positively correlated with changes in the average pollinator size rather than altitude.

Changes in pollinator communities can also drive plants away from investment in pollinator attraction and toward greater self-compatibility. For example, *Brassica rapa* plants became shorter with reduced floral volatiles and increased selfing under fly pollination compared to bumble bee pollination (Gervasi and Schiestl, 2017). Under low pollinator conditions, selection favors traits that promote autonomous self-fertilization, such as inward facing anthers and a reduced anther-stigma distance (Toräng et al., 2017). The shift from outcrossing to selfing is also associated with reduction in corolla size and an increase in pistil length (Carleial et al., 2017). Collectively, this work demonstrates that shifting insect communities are sufficient to drive evolution in plant traits.

### Land Use Change Affects Pollinators

The impact of land use change on plant–pollinator interactions in habitat fragments can have diverse outcomes (Table 2) and will depend on the traits of the pollinators as well as the surrounding habitat matrix. Human modification of the landscape alters resource availability and at the same time presents additional stressors (pesticides, novel pathogens, altered microclimate; see Goulson et al., 2015). Consequently, reduced pollinator abundance and diversity within natural habitat fragments has frequently been documented in human-dominated landscapes (Jennersten, 1988; Aizen and Feinsinger, 1994a; Steffan-Dewenter and Tscharntke, 1999; Steffan-Dewenter et al., 2001; Kormann et al., 2015; Wenzel et al., 2020). However, given that landscape composition affects pollinators through two potential mechanisms: (1) changes in population size and (2) distribution of pollinators among habitats, we expect that pollinator interactions with wild plants respond differently depending on the mechanism at play. For example, agricultural covers such as mass flowering crops may provide complementary or supplementary resources which subsidize pollinator populations (Westphal et al., 2003; Rundlöf et al., 2014) and bee visitation to wild plants in natural habitats (Holzschuh et al., 2016). During bloom, however, the cover of mass flowering crops is generally associated with lower pollinator abundance in semi-natural habitats (Holzschuh et al., 2016; Montero-Castaño et al., 2016).

**TABLE 2:**
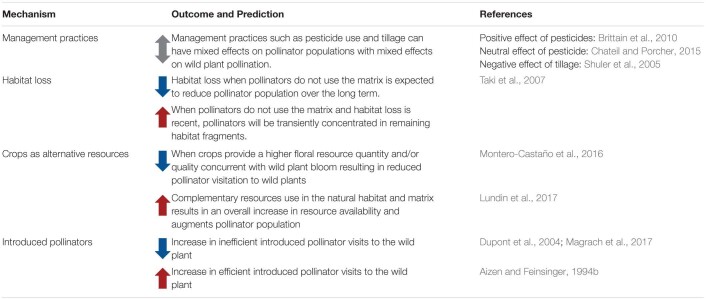
Effect of land use change (from natural to agricultural or urban areas) on pollinator communities and populations of wild plant systems.

*Here we present the direction of the response (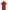 = positive effects on pollinators, 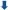 = negative effects on pollinators) and the potential mechanisms for that response. When examples from the literature are available that suggest those mechanisms, we cite them under references. Rows without references reflect areas of future work.*

Often the impact of land use change is not evenly distributed across pollinator communities. Instead, the traits of some pollinator groups allow them to persist in human-modified landscapes while other traits make some groups more vulnerable (Gámez-Virués et al., 2015; Wenzel et al., 2020). For example, Steffan-Dewenter et al. (2001) found no change in bumble bee visitation to a native plant across a semi-natural habitat gradient although solitary bee visitation declined. In this case, differences in responses may be due to differences in body size as large bodied bees are able to forage further (Greenleaf et al., 2007). Similarly, spillover of managed bees may substantially alter wild plant–pollinator interactions in human-dominated landscapes (Geslin et al., 2017). Both honey bees and managed bumble bees use a large proportion of non-crop pollen even when placed in crops during mass flowering (Foulis and Goulson, 2014; McArt et al., 2017) and can be more abundant than their wild counterparts in nearby natural habitats during periods of floral scarcity (González-Varo and Vilà, 2017; Trillo et al., 2019). For example, honey bee visitation to several native plants was highest in landscapes with low semi-natural cover while the opposite pattern was observed for wild bees (Steffan-Dewenter, 2002) suggesting that increased managed bee visitation may offset wild pollinator losses (Aizen and Feinsinger, 1994a).

Loss and fragmentation of natural habitat has frequently been correlated with reduced pollination and reproduction in wild plants (Aguilar et al., 2006). Because many pollinators utilize both wild plants and crops, sharing local pollinators creates potential fitness implications through interference in visitation rates or pollen transfer (Stanley and Stout, 2014). For example, mass blooming crops can mediate the spatial distribution of pollinators in the landscape, reducing visitation and fitness in co-blooming wild plants (Holzschuh et al., 2011; Van Reeth et al., 2019). Conversely, mass crop blooms may augment pollinator populations, increasing spillover and boosting the fitness of wild plants blooming immediately after the crop (Kovács-Hostyánszki et al., 2013). Therefore, mass flowering crops can have opposing outcomes for wild plant populations via separate mechanisms suggesting that the kind of habitat conversion as well as the traits of focal wild plant populations are likely to have strong impacts on the outcome of these land use changes on wild plant populations.

In some systems, plants demonstrate resilience to land use change. This is exemplified when there is no detectable effect of land use change on plant reproductive success (Lopes and Buzato, 2007; Ekroos et al., 2015; Skogen et al., 2016; Grass et al., 2018) likely because these landscapes select for pollinator species that are tolerant to disturbance and/or have a high dispersal ability. In restored prairies, increasing proportion of agriculture in the surrounding landscape did not increase pollen limitation in a pollinator dependent forb (Ritchie et al., 2020). It is possible that the restored prairies provide habitat to support sufficient pollinators to prevent pollen limitation. Further, conflicting pressures of pollinators and herbivores could also be responsible for the failure of these studies to detect a fitness effect of landscape composition (Steffan-Dewenter et al., 2001).

In summary, land use changes have substantial impacts on the composition of pollinator communities with potential consequences on plant fitness in remaining natural habitat fragments. As a rule, loss of natural habitat is expected to reduce the abundance and diversity of wild plant pollinators and consequently cause pollen limitation in wild plants (Taki et al., 2007); yet, exceptions to this rule, such as temporal effects of mass flowering crops, will alter these dynamics. Further, loss of natural habitat is also expected to shift flower visitor communities toward disturbance-adapted and managed-pollinator species.

### Land Use Change Affects Wild Plant Floral Traits

In spite of the evidence that land use change is altering pollinator communities and that these shifts can drive trait change in plants, there are few studies linking these two processes. There is some evidence that spatial variation in pollinator communities can mediate differential selection on floral traits (Ågren et al., 2013; Chapurlat et al., 2015). Irwin et al. (2018) linked pollinator mediated shifts in floral display size to land use change, with larger displays present in urban landscapes. Similarly, in scotch broom (*Cytisus scoparius*) pollinators are shown to impose selection for increased floral size in urban, but not rural landscapes (Bode and Tong, 2018). Further, in a study comparing pollinator mediated selection in insect-impoverished industrial sites and insect-rich natural habitats, plants in insect-impoverished landscapes produced smaller and fewer flowers and demonstrated higher potential for autonomous selfing than those in insect rich habitats (Brys and Jacquemyn, 2012). Overall, it seems that the effects of urbanization are inconclusive showing cases of increased, but also decreased investment in floral traits. Studies from agricultural land-use change seem to be completely missing today, leaving this field equally inconclusive. Here we present results from our own work examining the effects of agricultural land use change on flower size.

**FIGURE 4:**
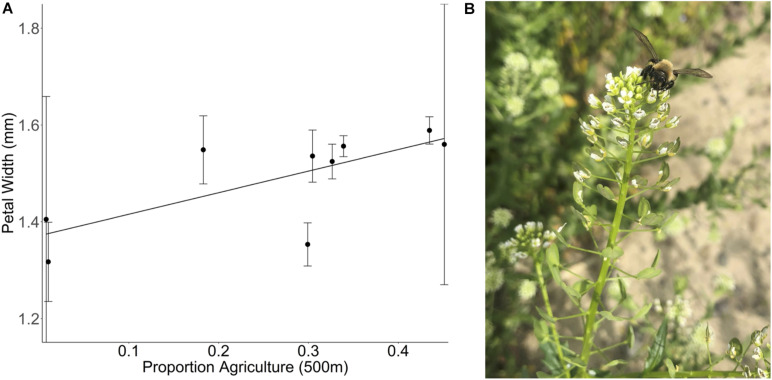
**(A)** Effect of the proportion of agricultural land at 500 m on the petal width of *Thlaspi arvense* plants. *T. arvense* plants were collected at nine different sites that vary in their landscape complexity [*F*_(__1_,_7__)_ = 5.5097, *p* = 0.0513]. The proportion of agricultural cover at a 500 m radius around the collection site is a measure of land use composition but does not provide any information regarding the intensity of the land management practices within any given land use. Mean ± 1 SE values per site are calculated based on the measurement of 157 flowers of 77 different *T. arvense* plants (2–15 plants per site). **(B)** A Melandrena visiting a *Thlaspi arvense* plant in the field.

In the summer of 2017, we collected seeds of the Brassicaceae species *Thlaspi arvense* from sites across a landscape gradient from low to high agriculture. Plants from each site were grown together in a greenhouse common garden and petal length and width was measured using electronic calipers (see Supplementary Methods). Plants sourced from areas with greater natural area produce smaller flowers than plants sourced from areas with very low natural area (Figure 4). This indicates increased investment in floral display in landscapes with a greater proportion agricultural cover as a potential response to compensate for changes in pollinator community assemblage or visitation rate given that flower size has been previously demonstrated to affect visitation by both small bees and syrphid flies in another brassica species (Conner and Rush, 1996). This trait adaptation could result from genetic change or maternal effects.

## Land Use Change Affects Indirect Defenses, Mediated by Natural Enemy Communities’ Responses to Land Use Change

### Natural Enemies Are Likely to Affect Plant Indirect Defenses

Plants express multiple traits that facilitate the host/prey search behavior or residence of natural enemies of herbivores, such as predators or parasitoids. These traits can indirectly increase resistance to herbivory and so potentially function as indirect defenses if the natural enemy attraction also results in relative increases in plant fitness. Experimentally demonstrating the effects of traits associated with natural enemy attraction on plant fitness has been difficult for experimental and biological reasons (Kessler and Heil, 2011). However, increased indirect resistance, the reduced performance or survival of herbivores mediated by plant traits attracting natural enemies, has been demonstrated commonly in multiple study systems (Dicke and Baldwin, 2010). Plant indirect resistance can be categorized into two groups based on the traits mediating it: resource-mediated indirect resistance and information-mediated indirect resistance (Kessler and Heil, 2011). Resource-mediated indirect resistance traits include physical structures (e.g., domatia, hollow stems and thorns) or food provisions (e.g., food bodies, extrafloral nectaries) that facilitate the residence time and abundances of natural enemies on plants (Heil et al., 2001; Llandres et al., 2019). Probably the most prominent example for such resource-mediated indirect defenses are ant plant–ant interactions, where plants provide both shelter, such as hollow stems and thorns, as well as food in the form of extrafloral nectaries and nutrient-rich food bodies. This form of indirect defense in an obligate relationship between ants and plants can be so efficient as a defense that the plants apparently lose their direct chemical defenses almost entirely (Heil et al., 1999, 2000; Dyer et al., 2001). This suggests a high functionality and efficiency of resource-mediated indirect defenses. And, indeed, such traits are taxonomically widely distributed in the plant kingdom (Beattie, 1989; Koptur, 1992). Nevertheless, demonstrating that these traits are functional as defenses requires demonstrating plant fitness differences that are associated with differential expression of those plant-derived resources via differential attraction of natural enemies and their effects on herbivory (Kessler and Heil, 2011). Other than in obligate ant plants, most of the natural enemy–plant relationships are facultative and depend on the availability of natural enemies in a given habitat. In one of the very rare cases that natural selection on one of the putative resource-mediated indirect defenses was measured, predatory ants were found as agents of selection on extrafloral nectar production of wild cotton, *Gossypium thurberi*, as nectar production correlated with reduced herbivory and increased plant fitness (Rudgers and Strauss, 2004). While questions about microevolutionary dynamics on resource-mediated indirect resistance traits remain largely open, their study along land use change gradients, is promising as such gradients provide a vast range of variation in herbivory (see above) as well as the availability of natural enemies of herbivores (see below). To our knowledge no such study on landscape-related expression of resource-mediated indirect defense traits and its ecological causes and consequences has been undertaken.

This is certainly similar for information-mediated indirect defenses. By far the best studied group of such traits is the herbivory-induced production of volatile organic compounds. The volatiles emitted from damaged plants can function as chemical information that facilitate the host/prey search behavior of natural enemies (predators or parasitoids) and so, presumably increase predation pressure on herbivores and, consequently, increase plant fitness (Dicke and Baldwin, 2010). It is probably one of the most widely distributed natural phenomena but there has yet to be a study to show evidence for natural selection on inducible volatile emission with predators as the major agents of selection (Kessler and Heil, 2011). Nevertheless, there is overwhelming evidence that differential VOC emission in response to herbivory results in differential attraction of natural enemies (Dicke and Baldwin, 2010). There is also plenty of evidence that this differential attraction has effects on herbivory (Kessler and Heil, 2011), especially when considering predators and egg parasitoids, collectively suggesting high resistance-mediating function on inducible volatile emissions. However, there are only few studies linking differential predator attraction and resulting changes in herbivory to plant fitness (Kost and Heil, 2008; Allison and Hare, 2009). In large part this is due to the fact that VOC-mediated indirect resistance is facultative, thus depending on the availability of natural enemies in the environment and subject to compromising interactions with other organisms in the environment that use the same chemical information. For example, herbivory-induced VOCs in *Brassica oleracea* do not only attract parasitoids, but also their hyperparasitoids, thus fourth-trophic level effects canceling out third-trophic level effects (Poelman et al., 2012). Similarly, the indirect effect of predation on herbivory mediated by the plant, makes more direct agents of selection on plant inducible VOC emission, such as herbivores and neighboring plants more likely. In a recent study on tall goldenrod, *Solidago altissima*, herbivory was identified as the agent of natural selection on the inducibility of VOC emission. In this case herbivore selection increased the plants’ ability to exchange chemical (VOC-mediated) information with their neighbors which allowed for a more even distribution of herbivory in the plant population and thus minimizing the damage each individual plant receives (Kalske et al., 2019). While this study supports the hypothesis that herbivory may be a more direct agent of selection on inducible VOC emission it also demonstrates that there can be strong selection on the inducibility of VOC emission. Thus, although natural enemies may not necessarily be the major agents of natural selection on inducible VOC emission, pronounced alterations in organismal interactions along environmental gradients may have significant consequences for the mean expression of information-mediated indirect defenses.

We do not currently know of any study investigating whether inducible VOC emission varies predictably along environmental gradients, such as those of land use change. The three characteristics of VOC bouquets that can vary are (I) strength of inducibility (e.g., increase in total emission), (II) induced changes in composition (e.g., relative production of certain compounds), and (III) changes in relative similarity of VOC bouquets among plants of the same population (Kessler and Shiojiri, 2016; Junker et al., 2017). The latter had been identified in a *Solidago altissima* study to increase information transfer between plants in populations of elevated herbivory and was interpreted as a convergence of chemical language (Kalske et al., 2019). A similar convergence or increased phenotypic integration was predicted for herbivore-induced VOC emission as functional attractants to predators and parasitoids. Interestingly, this increased phenotypic integration of herbivory-induced VOC emission in comparison to those of unchallenged plants has been found in a meta-analysis across a large number of study systems (Junker et al., 2017). Thus, when searching for signs of natural selection in association with altered herbivory and natural enemy availability in plant populations along environmental gradients, simple measurements of mean differences in inducibility of VOC emission may not be sufficient.

### Land Use Change Affects Natural Enemies

Although the direct effect of natural enemies on plant selection remains an area to be studied, there is clear evidence that land use change impacts natural enemy communities (Table 3). Land use change will negatively impact natural enemies, when the natural enemies cannot use the surrounding agricultural matrix. The surrounding matrix might not be suitable for natural enemies when it does not provide the resources necessary to support natural enemy populations due to host specialization, herbivore suppression (i.e., pesticide use), or lack of alternative resources such as nectar and pollen (Tscharntke et al., 2016). Additionally, anthropogenic management practices in intensively managed agricultural systems have been shown to reduce natural enemy populations through activities such as tilling, mechanical weed control, and broad spectrum insecticide sprays (Thorbek and Bilde, 2004; Nilsson, 2010; Rusch et al., 2011). Examples of negative effects of land use change have been reported for native lady-beetles in prairies (Werling et al., 2011), for specialist parasitoids of nettle aphids (Rand and Tscharntke, 2007), and for bark beetles and their natural enemies (Ryall and Fahrig, 2005).

**TABLE 3:**
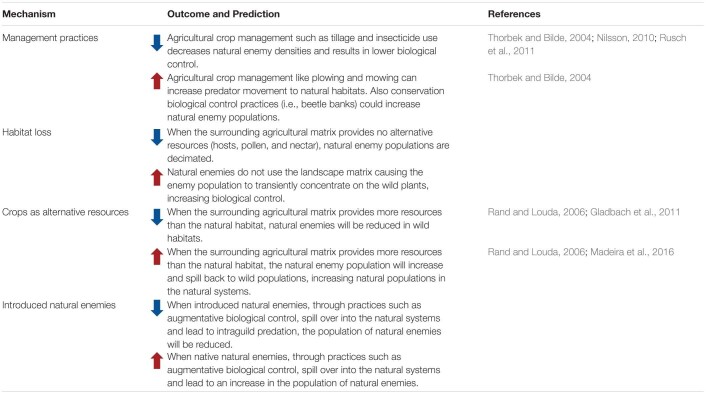
Effect of land use change (from natural to agricultural or urban areas) on natural enemy communities and populations of wild plant systems.

*Here we present the direction of the response (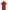 = positive effects on herbivores, 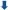 = negative effects on herbivores) and the potential mechanisms for that response. When examples from the literature are available that suggest those mechanisms, we cite them under references. Rows without references reflect areas of future work.*

Interestingly land use change can differentially affect natural enemies depending on their extent of specialization and invasiveness. For example, in prairie systems in Michigan and Wisconsin, the abundance of native lady-beetles decreased, while the abundance of exotic lady-beetles did not change with increasing annual agricultural cover (Werling et al., 2011). For nettle aphids, specialist parasitoids were reduced by land use change, while generalist predators increased with an increase in the surrounding agricultural matrix (Rand and Tscharntke, 2007).

An increase in natural enemy abundance related to land use change is expected to occur when the surrounding agricultural matrix provides more resources such as hosts, overwintering sites, pollen and nectar to natural enemies than natural habitats (Tscharntke et al., 2016). For example, the irrigation of crops in dry regions or seasons can provide resources to herbivores and natural enemies at times when the natural habitats are inhospitable or have a low productivity (Costamagna et al., 2015).

The scenarios in which natural enemies prefer and thrive in agricultural matrices could lead to permanent or temporarily different outcomes for wild plants. If natural enemies prefer agricultural fields over adjacent natural areas, there could be a reduction in natural enemies and biological control for wild plants. This reduction could be permanent or limited in time if these natural enemies spill back and even increase the number of natural enemies in natural areas. Empirical work has shown that the adjacent crop habitat (Madeira et al., 2016), and management practices such as harvesting (Schneider et al., 2016), mowing and plowing (Thorbek and Bilde, 2004) can increase the movement of natural enemies from agricultural areas back into natural systems. Also, natural enemy traits and the availability of hosts have been shown to affect the intensity of the spillover effects, as was shown by Frost et al. (2015). Another mechanism through which land use change can increase natural enemies in natural systems, is through management practices, such as augmentative biological control. As with herbivores and pollinators, natural enemies introduced through agriculture can disrupt enemy–prey interactions in natural habitats (Snyder and Evans, 2006). However, that disruption can have very different consequences depending on if the natural enemies mostly control the herbivores, or if they reduce the local natural enemy population through intra-guild predation (Snyder and Evans, 2006).

Landscape-mediated increases on natural enemies have been shown to translate into reduction of herbivores (Balzan et al., 2016). For example, in grassland remnants in Nebraska, an increase in the surrounding agricultural land cover led to an increase in the abundance of lady beetles and a higher suppression of aphids on wild plants (Rand and Louda, 2006). Similarly, in woodland fragment edges adjacent to roads and residential areas pupa parasitism of the holly leaf miner, *Phytomyza ilicis*, was higher than in woodland interiors (McGeoch and Gaston, 2000). This would suggest that natural plants at sites that have experienced land use change might benefit from the presence of natural enemies and could be investing more in indirect defenses to take advantage of the higher abundance of natural enemies in the community.

### Land Use Change Affects Plant Indirect Defenses

Although we were not able to find any studies that have directly measured the effect of land use change on plant indirect defenses in natural plant populations, the little evidence we have suggests that there could be differential selection by natural enemies on indirect defense traits. In a study done on cabbage infested with herbivore *Pieris brassicae* placed in different landscapes, it was found that the induction of plant volatiles in response to herbivory can mediate the effect on parasitism (Aartsma et al., 2020). To test this, two different accessions of cabbage were used. One accession was more attractive to the parasitoid *Cotesia glomerata* while the other accession was less attractive to the same parasitoid. The attractiveness is thought to be mediated by differences in herbivore induced plant volatile blends, that have been found to be different between these two accessions (Poelman et al., 2009; Aartsma et al., 2019). As the proportion of arable land cover increased in the landscape (at a 200 m radius) the parasitism rate of the caterpillars increased. The increase in parasitism was stronger for the more attractive variety than for the variety that was less attractive. With low agricultural cover there was no difference in parasitism rate, but with higher agricultural cover, where parasitism was also higher, the attractive variety also had significantly more parasitism. This example shows that parasitoids in different landscapes are responding differently to indirect defense traits on plants, and although the plants used are cultivated plants, we could expect that the effect is similar for natural plant populations. Further work looking at the importance of indirect defense traits in plants along human-made land-use change gradients, would give us more insights into the importance of these effects.

## Implications for Complex Plant-Mediated Interactions

Although in this review we have addressed the evolutionary consequences of each insect group on plant traits separately, in reality the landscape context influences all insect groups simultaneously yet not necessarily in the same way. Further, many plant traits mediate interactions across different insect guilds, while also changing how insects interact with each other. These complex relationships determine the outcomes of interactions and thus the effects on plant growth and reproduction. In consequence, the interactor that has the strongest effect on plant fitness in an isolated two-species interaction may not be the major agent of selection on associated traits. For example, landscape-mediated increases in herbivory and the associated variation in the proportion of plants damaged and induced by one herbivore can make plants more susceptible or more resistant to other herbivores, which would be reflected in the relative compositional changes of the herbivore community (Kessler and Baldwin, 2004; Viswanathan et al., 2005). This can even mean that the presence of a less damaging herbivore that reduces the impact of a more damaging herbivore confers a net fitness benefit to the plant (Kessler and Baldwin, 2004), with the less damaging herbivore becoming a keystone herbivore and the strongest agent of selection on the interaction-mediating plant traits (Poelman and Kessler, 2016).

Different types of defenses can interact with each other and so dramatically affect the outcome of interactions. One of the most cited examples is the interaction of plant endogenous signaling pathways when plants respond to herbivores and pathogens, which in many cases have been shown to elicit conflicting responses (Thaler et al., 2012). When strictly considering interactions among arthropods, direct defenses can function synergistically with indirect defense traits, whereby induced direct resistance can reduce the growth and defensibility of herbivores and so expose them more to natural enemies that are simultaneously attracted by herbivory-induced volatile emissions (Kessler and Baldwin, 2004; Fatouros et al., 2014). In contrast, plant direct defenses can also reduce the survival of natural enemies as they may be directly exposed to defense metabolites on the plant surface or indirectly to plant toxins that have been sequestered by specialist herbivores (Dyer et al., 2001; Ballhorn et al., 2008). In addition, herbivore-induced VOC emissions may not only attract natural enemies of herbivores but also predators and parasitoids of the predators so erasing the indirect defense effect (Poelman et al., 2012).

One of the complex community interactions that can be particularly impactful for plant trait evolution is the plant-mediated interactions between herbivores and pollinators. As pollinators, just like herbivores, consume plant products, they are similarly exposed to plant defenses, which results in a conflict for the plant to attract mutualist pollinators while repelling antagonistic herbivores (Kessler and Chautá, 2020). Mechanisms to overcome this conflict include antagonistic pleiotropy between defense and reproductive traits altering resource allocation into and secondary metabolite concentrations in floral rewards, or shifted flowering phenology (Mothershead and Marquis, 2000; Lucas-Barbosa et al., 2016; Jacobsen and Raguso, 2018). In tobacco, *Nicotiana* spp., species with greater dependency on pollinators produce lower amounts of defense-related alkaloids in comparison to species that do not rely on animal pollination (Adler et al., 2012). In a study with a broader taxonomic scope across the Solanaceae plant family, the phylogenetic shift from self-incompatibility (e.g., strong dependency on animal pollinators) to self-compatibility was associated with decreased constitutive resistance and increased inducibility of resistance (Campbell and Kessler, 2013). Such a linkage between defense and reproductive strategy on the macro-evolutionary level seems to be reflected in microevolutionary dynamics as well. For example, in *Brassica rapa*, exposure to selection by bee pollinators increased mean floral attractiveness. However, the additional presence of herbivores in the population selected for reduced separation of stigmas and anthers (herkogamy) and the associated increase in self-compatibility and autonomous selfing. This suggests that altered selection by herbivores can strongly affect plant defense expression and so the interaction of plants with pollinators (Ramos and Schiestl, 2019). On an ecological scale plant induced responses to herbivory can result in pollinator limitation that can either be compensated for by changes in plant phenology (Schiestl et al., 2014) or mating system (Kessler and Halitschke, 2009) or result in apparent ecological fitness costs (Kessler et al., 2011). In some cases, insects may function as ‘pollinating-herbivores,’ shifting from larval herbivore to adult pollinator as they progress through life stages (Altermatt and Pearse, 2011). This creates further conflict as plants must defend against damage while also cultivating pollinators and maintaining pollinator attraction (Sharp et al., 2009; Lucas-Barbosa, 2016). Ultimately, as any one insect is affected by land use change, the consequences may cascade through this network of interactions altering how insects interact with plants and each other. Previous work has emphasized the effects of land use change on whole communities, yet insect functional traits are also affected by land use change. In pollinators for example, land use change can decrease body size (Persson and Smith, 2011; Renauld et al., 2016; Grab et al., 2019), which can negatively affect the pollination services provided to plants (Jauker et al., 2016). While landscape mediated functional trait evolution is not well studied, we expect these trait expression shifts are present across insect groups and important in mediating insect interactions with plants. However, the nuances of insect functional trait evolution go beyond the scope of this paper.

**Box 1. Proposed Future Work.**

1. *Link land use change to the evolution of plant traits.* In a single study test all three steps highlighted in this review: (1) land use affects an insect interactor, (2) the interactor affects the plant traits, and (3) land use affects the plant traits via changes in the interactor. These studies would need to be repeated for multiple systems and sites to answer the following question: Are there broad patterns across systems and insect groups?
2. *Eco-evolutionary feedback loops in plant communities:* Determine how changes in plant traits lead to an eco-evolutionary feedback loop between the insect community and the plant community driving change beyond the plant population.
3. *Land use change and plant indirect defenses.* How does land-use change affect plant indirect defenses? Is there conflicting selection on plant indirect and direct defenses or do they function synergistically? What is the role of abiotic factors like pesticides and fertilizer in mediating the relationship between plants and natural enemies?
4. *Land use change and plant population genetics.* How does land use change affect plant populations genetics? To what extent can variation be explained by plasticity and maternal effects? Further, selection experiments along the land-use change gradients are equally important to determine if these changes in traits are adaptive.

Though we have highlighted numerous challenges in disentangling plant-insect interactions across landscapes, we present land use gradients as a unique opportunity to use modified landscapes as a natural experiment. The magnitude of land use change creates an experimental framework that can be utilized in systems across the globe. Experiments within this framework can reveal the selection pressure exerted by different organisms, the resulting effect on plant fitness, and how the relative importance of each interactor changes with landscape mediated changes in insect community composition. We are not aware of any one study that simultaneously examines the following three steps linking land use change to the evolution of plant traits via some insect interactor: (1) land use affects the interactor, (2) the interactor affects the plant traits, and (3) land use affects the plant traits via changes in the interactor. While descriptive studies are a crucial starting point, selection experiments are needed to establish a causal link and to test the role of maternal effects. We propose artificial selection experiments in which individuals from the extreme ends of the land use gradient are crossed and the offspring self-pollinated to segregate traits. The large variation in traits of these F2 populations can be used to evaluate which traits are selected for in given environments and which insect interactors are likely important agents of selection. Pollinator and herbivore exclusion treatments would be necessary to disentangle the role of each group. Further, abiotic factors associated with land use change such as pesticide and fertilizer runoff are also known to impact wild plant communities (Didham et al., 2015; Botías et al., 2016). These factors may act as additive or conflicting forces with insect-imposed selection and thus are also important to quantify. We present what we consider the most important questions we think should be resolved next to better understand plant-insect evolutionary dynamics (Box 1).

## Final Conclusion

The conversion of natural habitats to human-modified landscapes disrupts the stable species interactions that evolved in these landscapes. Unlike natural disturbances where succession restores many pre-disturbance species interactions, human-modified landscapes are maintained in a modified state. Through this review and our own results, we have collected evidence suggesting that altered communities of pollinators, herbivores, and natural enemies occupy these modified landscapes and that each of these insect groups can act as a selective force. Thus, we hypothesize that wild plants are experiencing differential selection based on landscape context through interactions with the resulting community of insects. Further studies are needed to examine the outcomes of landscape-mediated selection on individual plant traits and ultimately to understand the consequences for wild plant communities on a broader scale.

Differential selection on plant traits across the landscape likely has cascading consequences for population and community dynamics driven by evo-eco feedback loops. In each landscape context, the availability of insect interactors depends on whether an insect is able to utilize the modified habitat. Plant trait evolution and changing plant community and population dynamics, in turn, will affect which members of the insect community remain or are added to interact with the plant community. Selection on traits that increase or decrease the attractiveness of a plant population to herbivores and/or pollinators may impact the entire plant community, potentially altering the insect community drawn to a patch and the relative competitive ability of plants within the patch. This may create a feedback loop where plant adaptation reinforces traits in the insect community and patterns of insect availability. There is already evidence that human-mediated land use change is linked to trait filtering in plants and insects, and selection by insects may be one additional mechanism through which this trait filtering is occurring in plants (Gámez-Virués et al., 2015; Mendes et al., 2016; Boukili and Chazdon, 2017).

Through this review we hope to motivate further studies examining the potential for wild plants to adapt with land use change, the outcomes of conflicting selection, and the ultimate consequences for plant fitness and community structure. Future work should examine plant population genetics to establish if observed phenotypic changes represent plasticity or genetic change. Additionally, while many studies suggest the mechanisms by which land use change affects natural plant communities, few actually test these claims. Of all the patterns examined here, the effects of land use change on indirect defenses remains the least studied (Box 1). Given that human-modified habitats dominate the global landscape, understanding how wild plants in these landscapes are adapting and how their communities are changing could help inform which species are the most vulnerable. Human-initiated land use change represents an unintended experiment in plant evolution on a global scale, creating opportunity to expand basic research in plant adaptation and evo-eco dynamics, and potentially guide conservation action.

## Data Availability Statement

The datasets presented in this study can be found in online repositories. The names of the repository/repositories and accession number(s) can be found below: https://doi.org/10.5061/dryad.ncjsxkst4. The data is uploaded to dryad but not publicly available while under review.

## Author Contributions

KP and AK conceived the idea of the manuscript. HG and KP collected the data. HS and HG analyzed the data. HS drafted the manuscript to which all authors contributed sections and revisions.

## Conflict of Interest

The authors declare that the research was conducted in the absence of any commercial or financial relationships that could be construed as a potential conflict of interest.
