# Supplementary material for: Human-Mediated Land Use Change Drives Intraspecific Plant Trait Variation
Source: Front Plant Sci. 2021 Jan 14;11:592881. doi: 10.3389/fpls.2020.592881 (PMC7840540; doi:10.3389/fpls.2020.592881)
Supplement: Supplementary file 1 [file Table_1.docx]

**Human mediated land use change drives intraspecific plant trait variation**

Supplemental Materials

Methods for data presented in figures 1, 3 and 4

Two wild brassica species (*Barbarea vulgaris* and *Thlaspi arvense*) were collected in July, 2017 and one species (*Capsella bursa-pastoris*) in July, 2019 from the field margins of farms (hereafter called sites) in the Finger Lakes Region of Upstate New York. We collected *B. vulgaris* from 10 sites, *T. arvense* from 9 sites and *C. bursa-pastoris* from 15 sites. Seeds from each site were germinated in petri dishes and then individually planted in 4-inch pots in a greenhouse common garden in the fall of 2017 for *B. vulgaris* and *T. arvense* and the fall of 2019 for *C. bursa pastoris*. These experiments provide preliminary data intended to spur more rigorous examination of the hypotheses presented in this review. We do not intend for the data provided to stand alone.

**Defense traits**: In the fall of 2017, we conducted a caterpillar bioassay on *B. vulgaris*. Plants were sourced from 10 sites, with 8-10 plants grown from each site (apart from one site in which there were only two replicates due to low germination). Two leaves were collected from each plant and placed in individual floral water tubes. Each tube was then inserted into a plastic lid and covered with a plastic cup to create a rearing chamber. Two three day old *Trichoplusia ni* caterpillars (sourced from Benzon Research) were placed on each leaf and allowed to feed for three days. At the conclusion of the bioassay, we recorded the mass of each surviving caterpillar. Caterpillars experienced 14% mortality across the experiment. Interestingly, of the two caterpillars placed on an individual leaf, across all replicates one caterpillar developed normally while the other’s growth was stunted. In our analysis of caterpillar growth, we used only the caterpillar that was successful on each leaf.

**Floral traits**: In December 2017, *T. arvense* plants were vernalized in a refrigerator at 4ºC and returned to the greenhouse early in 2018. Upon blooming, we measured petal width using handheld calipers as a proxy of flower size. Flowers were measured each week during the blooming period so that 1-3 flowers were measured per plant depending on the length of the bloom. Overall, 158 *T. arvense* flowers were measured representing 2-15 plants per site.

**Self-Compatibility**: *C. bursa-pastoris* seeds planted in August 2019 bloomed without vernalization in October and November. Each plant was allowed to self-pollinate with no hand pollination. After the plants finished blooming, we collected and weighed the seeds from the first 15 non-aborted pods.

**Statistical Analysis**: For both the floral and resistance traits, we conducted our analysis in R (R version 4.0.2) using the nlme package (Pinheiro et al., 2014) to build mixed effect models. For resistance traits, we used caterpillar end mass as the response variable and the proportion agriculture at 1500m as the predictor variable. For floral traits, petal width was used as the response variable and the proportion agriculture at 500m as the predictor variable. To analyze self-compatibility, we used seed mass as the response variable and the proportion of natural land cover at 1000m as the predictor variable. We included site and plant as a random effect to avoid pseudoreplication given that we had several plants per site and replicate samples from each plant.

References

Pinheiro, J., D. Bates, S. DebRoy, D. Sarkar, and R Core Team. 2017. nlme: Linear and

nonlinear mixed effects models. R package version 3.1-131. [https://CRAN.R-](about:blank)

project.org/package=nlme
